# Estimation of the Serum Levels of Brain-Derived Neurotrophic Factor (BDNF) and Interleukin-1β and Their Correlation With Clinical Severity in Depression Patients: A Case-Control Study

**DOI:** 10.7759/cureus.89261

**Published:** 2025-08-02

**Authors:** Chandrashekar B Huded, Supriya Yedve, Harish G Bagewadi, Navin Satyanarayan, Renuka S Melkundi, Prabhukiran V Gogi, Shivalee A, Rohini Kallur

**Affiliations:** 1 Psychiatry, Gulbarga Institute of Medical Sciences, Kalaburagi, IND; 2 Psychiatry, Faculty of Medical Sciences, Khaja Bandanawaz (KBN) University, Kalaburagi, IND; 3 Pharmacology, Gulbarga Institute of Medical Sciences, Kalaburagi, IND; 4 Biochemistry, Gulbarga Institute of Medical Sciences, Kalaburagi, IND; 5 Otolaryngology, Gulbarga Institute of Medical Sciences, Kalaburagi, IND; 6 Research, Gulbarga Institute of Medical Sciences, Kalaburagi, IND

**Keywords:** bdnf, biomarkers, depression, elisa, il-1β

## Abstract

Background: Depression is a complex psychiatric disorder with no universally accepted risk assessment tools. The pathogenesis of depression remains incompletely understood, and the exact mechanisms underlying its onset are still debated. A prevailing hypothesis suggests that dysregulated cytokine production, driven by immune system hyperactivation, may play a role in the development of depression.

Methods: This study aimed to investigate alterations in serum levels of brain-derived neurotrophic factor (BDNF) and Interleukin-1β (IL-1β) in individuals with depression and assess their potential roles as biomarkers. This was a case-control study. Cases diagnosed with depression were compared with healthy controls. The severity of depression was assessed using the Hamilton Depression Rating Scale (HDRS). Serum levels of BDNF and IL-1β were quantified using enzyme-linked immunosorbent assay (ELISA) kits procured from MyBioSource company.

Results: Patients with depression exhibited significantly higher serum levels of IL-1β and lower levels of BDNF compared to healthy controls. Moreover, a significant positive correlation was found between IL-1β levels and HDRS scores, while BDNF levels showed a significant negative correlation with HDRS scores.

Conclusion: Our findings suggest that serum IL-1β levels are positively correlated, while BDNF levels are negatively correlated, with the severity of depression. These biochemical markers may serve as potential risk assessment indicators and biomarkers for depression, providing insight into the underlying mechanisms of the disorder.

## Introduction

Depression is a widespread and persistent mental health disorder that leads to significant disability and increased mortality worldwide. While antidepressant medications are essential for treatment, gaining a deeper understanding of the underlying mechanisms of depression, especially the involvement of inflammation, is very critical in identifying newer therapeutic approaches. Major depressive disorder (MDD) is influenced by various factors, including genetic variations, neurotransmitter imbalances, chronic stress, and structural alterations in the brain. Recent investigations highlight the involvement of immune system components, such as cytokines, chemokines, neurotrophins, and growth factors, in the development of depression, though the precise causal pathways are still being explored. Although proinflammatory cytokines have been linked to MDD, the connection between peripheral cytokine levels and the disorder has not been definitively established [1].

The neurotrophic hypothesis suggests that reductions in brain-derived neurotrophic factor (BDNF) and other growth factors contribute to the degeneration of neurons in key regions like the hippocampus and prefrontal cortex. It is believed that antidepressants exert their effects by enhancing BDNF expression, thereby supporting neuroplasticity. Evidence from post-mortem studies has indicated lower levels of BDNF in the brains of suicide victims, lending support to this hypothesis [2]. Furthermore, proinflammatory cytokines, such as (IL-1β) Interleukin-1 beta, play a critical role by activating the hypothalamic-pituitary-adrenal axis and stimulating cortisol release, which exacerbates inflammation and creates a cycle of neurotoxicity that may perpetuate depressive symptoms. Elevated IL-1β has been linked to various depressive symptoms, including anhedonia and memory impairments, while inflammatory processes can hinder neurogenesis in the hippocampus [3].

Moreover, treatment with antidepressants has been found to decrease IL-1β levels in individuals with depression [4]. Several investigations have examined the relationship between cytokines and depression, demonstrating that inhibiting cytokines, particularly IL-1β, can reduce depressive symptoms and enhance neurogenesis [5]. Given the interconnection between pro-inflammatory cytokines, BDNF, and the severity of depression, these biomarkers are likely to influence the progression of the disorder. However, few studies have thoroughly explored the interactions between neurotrophic factors and inflammatory biomarkers in the development of MDD [3-5]. The present study aims to assess and compare the serum concentrations of BDNF and IL-1β in individuals with depression, helping to identify potential biomarkers that could predict disease progression and responses to treatment.

## Materials and methods

This was an interventional case-control study. The study was carried out at the Department of Psychiatry. The study was conducted from October 2023 to October 2024. The prior approval was taken from the Institutional Ethics Committee before the conduct of the study. The IEC approval number is GIMS/KLB/PHARMA/IEC/206/2023-24.

Inclusion criteria

Patients of either sex, aged between 19 and 65 years, who met the DSM-5 criteria [6] for depression were included. Apparently healthy controls aged between 19 to 65 years were matched.

Exclusion criteria

Patients with dyslipidemia, cerebrovascular event, hypertension, atherosclerotic disease, diabetes mellitus, and chronic inflammatory conditions [7]. Patients with a history of substance abuse, neurological disorders such as dementia, seizures, stroke, pregnant or lactating women, and medication intake for neuropsychological disorders were excluded from the study [8].

In this study, a total of 60 subjects were recruited. Patients diagnosed with depression (n = 30) and apparently healthy controls (n=30) aged between 19 and 65 years inclusive of both sexes were matched.

Sample size calculation was performed in the biostatistics department in our medical college. Calculations were done by the Epi Info, a program version 7.1 developed by the Center for Disease Control and Prevention [9]. The study considered 5% margin of error, with 4% prevalence rate of depression in a previous study done by Arvind et al. [10]. In this study, the confidence level was set at 95%, with the % of controls exposed as 60% and an odds ratio of 10. The ratio of controls to cases is 1, with a power of 80%, and an α value of 0.05. This was in accordance with a previous study done by Karampudi et al. [11]. By substituting the values, we calculated a sample size of 30 cases and 30 controls. So, p= 0.04; (1-p) = 0.96, Z score of 1.96 and d = allowable error (% in this study) = 0.05. By substituting the values in the standard statistical formula [12], N = Z2 x p x (1-p)/d2. By substituting the values, the sample size (n) came to be 60. The Hamilton Depression Rating Scale (HDRS) score is one of the most widely used scales to measure the severity of depression done clinically by using a 17-item questionnaire which originated in 1960 and treated as the gold standard [13]. Scoring is done based on the severity of symptoms where 0 being no symptoms. Based on the final score, the overall severity of depression was measured, <7 as normal, 8-13 as mild, 14-18 as moderate, 19-22 as severe, and 23 as very severe depression [14].

Blood collection and measurement of BDNF and IL-1β levels and HDRS scores were done for each subject. By means of venipuncture, 5 ml of blood was withdrawn from study participants. The blood was placed in plain tubes. Centrifugation of the sample was done at 3000 rpm/ 10 minutes. At -20^o^C, the serum was stored for the analysis of BDNF and IL-1β levels [12]. In the serum BDNF, IL-1β levels were determined by using commercially available ELISA kits procured from MyBioSource company.

The serum BDNF concentrations were determined by procuring the human from MyBioSource company bearing Catalog No.-MBS700602. The BDNF ELISA kit had a sensitivity of less than 0.063 ng/ml. The detection range was- 0.3125 ng/ml to 20 ng/ml.

Reproducibility

Both intra-assay CV (%) and inter-assay CV (%) were less than 12%. The serum Interleukin-1β concentrations were determined by procuring the human Interleukin-1β ELISA kits from MyBioSource company bearing Catalog No.-MBS263843, having sensitivity up to 5 pg/ml. The detection range was 15.6 pg/ml-1000 pg/ml.

Statistical methods for analysis of data

The Social Sciences (SPSS) software version 16 (SPSS Inc., Chicago, USA; Released 2007) was used for the statistical analysis [11]. The quantitative variables were expressed as mean ± standard deviation. The qualitative variables were expressed as counts and percentages [11]. Student’s t-test was used to compare all the parameters in the two groups. The chi-square test was used to compare the discrete covariate parameters. Pearson’s correlation coefficient was used to analyze the correlation between variables [12]. The p-value ≤ 0.05 was considered statistically significant.

## Results

In Table 1, the study participants were 30 cases and 30 controls. The mean ages of patients with cases and controls were 47.7±9.49 and 47.8±9.28 years, respectively. The chi-square test was applied. There was significant statistical significance of p<0.05. There were 66.7% non-educated participants in the case group versus 56.6% in the control group. There were 10% of participants with a history of substance abuse in the case group versus 5.0% in the control group; 73.3% participants were unemployed in the case group versus 53.4% in the control group, respectively. Also, the cases showed a BMI of 34.8±3.72, whereas controls showed a BMI of 26.2±2.44, which was statistically significant p*< 0.05.

**Table 1:** Comparison of the socio-demographic profile and laboratory parameters between the case and control groups. Data are presented as frequency, percentages, mean, and standard deviation. The chi-square test was applied. p-value with * indicates statistical significance (p<0.05). BMI: Body mass index; n: number

Socio-demographic Parameters	Cases (n= 30)	Control (n = 30)	p-value
Age (years)	47.7±9.49	47.8±9.28	0.989
Education- Educated/ Non-educated	10:20 (66.7%)*	13:17 (56.6%)	< 0.05
History of substance abuse-alcohol, smoking, tobacco chewing-	04 (10.0%)*	2 (5.0%)	< 0.05
Female: Male	13: 17(56.6)*	16: 14	0.052
Employment- Employed/unemployed	08:22 (73.3%)*	14:16 (53.4%)	< 0.05
BMI	34.8±3.72*	26.2±2.44	0.042

In Table 2, it is observed that serum BDNF levels in depressed patients were 4.08±2.48 ng/ml, which were significantly lower than those in the control group (5.42±2.14 ng/ml, p<0.01). Similarly, serum IL-1β levels were significantly higher in depressed patients (26.20±8.75 pg/ml) compared to the control group (17.71±7.15 pg/ml, p<0.01).

**Table 2:** Comparison of levels of BDNF and IL-1β in the case and control groups. Data are presented as mean and standard deviation; Student’s t-test was applied. p-value with † indicates statistical significance (p<0.01). BDNF: Brain-derived neurotrophic factor; IL-1β: interleukin-1 beta; n: number

Variables	Cases (n= 30) Mean ± SD	Control (n = 30) Mean ± SD	p-value
BDNF (ng/ml)	4.08 ± 2.48^†^	5.42 ± 2.14	< 0.01
(IL-1β) (pg/ml)	26.20 ± 8.75^†^	17.71 ± 7.15	< 0.01

In Table 3, it is observed that serum BDNF levels in depressed patients with HDRS scores ≥15 were 3.42±1.26 ng/ml, which showed a statistically significant decrease (p<0.05) when compared to those with HDRS scores ≤ 15 of 4.64±1.48 ng/ml. Similarly, it is observed that serum IL-1β levels in depressed patients with HDRS scores ≥15 of 32.20±7.64 pg/ml, which showed a statistically significant increase (p<0.01) when compared to those with HDRS scores ≤15 of 27.60±5.52 pg/ml.

**Table 3:** Table presenting the depressed patients grouped into two groups based on HDRS scores. A total of 22 patients had an HDRS score ≥ 15, while eight patients had a score ≤ 15. Data are presented as mean and standard deviation; Student’s t-test was applied. p-value with * indicates statistical significance (p<0.05). p value with † indicates statistical significance (p<0.01) BDNF: Brain-derived neurotrophic factor; IL-1β: Interleukin-1 beta; HDRS: Hamilton Depression Rating Scale; n: number

Variables	HDRS score ≥ 15 (n = 22) Mean ± SD	HDRS score ≤ 15 (n = 8) Mean ± SD	p-value
BDNF (ng/ml)	3.42±1.26^*^	4.64±1.48	0.044
(IL-1β) (pg/ml)	32.20±7.64^†^	27.60±5.52	0.010

In Table 4, it is observed that there was a statistically significant (p<0.001) negative correlation between BDNF levels and HDRS scores with a Pearson coefficient (r) value of (-0.562) and a statistically significant (p<0.001) positive correlation between IL-1 β levels and HDRS scores with a Pearson coefficient (r) value of (+0.743).

**Table 4:** Correlation between HDRS scores, BDNF, and IL-1β levels. Student’s t-test was applied. p-value with†† indicates statistical significance (p< 0.001). (r) value: Pearson coefficient BDNF: Brain-derived neurotrophic factor; IL-1β: Interleukin-1 beta; HDRS: Hamilton Depression Rating Scale

Correlation	(r) value	p-value
BDNF and HDRS scores	- 0.562^††^	< 0.001
IL-1β and HDRS scores	+ 0.743^††^	< 0.001

In Table 5, it is observed that serum BDNF levels in depressed patients with >2 stressful life events were 3.12±1.38 ng/ml which showed a statistically significant decrease (p<0.05) when compared to those with scores ≤2 stressful life events of 3.84±1.26 ng/ml. Similarly, it was observed that serum IL-1β levels in depressed patients with>2 stressful life events were 33.45±7.89 pg/ml which showed a statistically significant increase (p<0.05) when compared to those with scores ≤2 stressful life events of 26.19±4.21 pg/ml.

**Table 5:** Relationship between the number of stressful life events and serum BDNF and IL-1β levels in patients with depression. Data are presented as mean and standard deviation; Student’s t-test was applied. p-value with * indicates statistical significance (p<0.05). BDNF: Brain-derived neurotrophic factor; IL-1β: Interleukin-1 beta; HDRS: Hamilton Depression Rating Scale; n: number

Variables	Depression with>2 stressful life events (n=18) Mean ± SD	Depression with ≤2 stressful life events (n=12) Mean ± SD	p-value
BDNF (ng/dl)	3.12±1.38*	3.84 ±1.26	0.026
IL‑1 β (pg/ml)	33.45±7.89*	26.19±4.21	0.018

## Discussion

The present study investigates the role of biological markers, specifically serum BDNF and IL-1β levels, in patients with depression compared to healthy controls. While the pathogenesis of depression remains incompletely understood, emerging evidence suggests that low-grade systemic inflammation plays a critical role in its development.

Our findings showed that most patients with major depressive disorder (MDD) were males (56.6%), unemployed (66.7%), and had substance abuse (10 %), consistent with previous studies by Farid et al. [15] and Yang et al. [16]. This demographic pattern may be influenced by factors such as smoking, obesity (BMI ≥30 kg/m²). In the present study, the BMI among cases was 34.8±3.72 when compared to the controls was 26.2±2.44, which was statistically significant and was associated with increased depression risk in a similar study [17]. The BDNF, a crucial neurotrophic factor involved in neuronal survival, synaptic integrity, and plasticity, has been shown to be significantly reduced in untreated depressed patients compared to healthy controls or successfully treated patients [18]. In our study, we observed the serum BDNF levels in depressed patients to be 4.08±2.48 ng/ml, which were significantly lower than those in the control group (5.42±2.14 ng/ml, p^†^<0.01) which align with prior research done by Karege et al. [19] who reported a negative correlation between BDNF levels and depression severity.

Similarly, other studies have highlighted the relationship between lower BDNF levels and depression recurrence and severity [20]. Our study showed a statistically significant negative correlation between serum BDNF levels and the severity of depression, contrary to some earlier studies. In a previous study, it was observed that BDNF levels were increased significantly after antidepressant treatment [21]. This discrepancy may be attributed to individual variability in depression pathophysiology or differences in the methods used to assess depression severity. BDNF’s role in neuronal plasticity may influence the recurrence or recovery of depressive episodes, and future research should explore its potential as a therapeutic target [7].

Regarding inflammation, our study observed significantly elevated serum IL-1β levels in depressed patients, which is consistent with previous research indicating that MDD is often accompanied by an immune response involving T-lymphocytes and monocytes/macrophages [22]. The previous study by Brietzke et al. [23] also found a positive correlation between mood symptoms and IL-1β and IL-6. Chronic stress, associated with HPA axis overactivity and elevated cortisol levels, may lead to the elevation of inflammatory markers, which could exacerbate depression [7]. In our study, the mean age of patients with cases and controls was 47.7 ± 9.49 and 47.8 ± 9.28 years, respectively, as shown in Table 1, which was a similar finding in the previous study [24]. Moreover, gender differences, such as the immunosuppressive effects of testosterone versus the immune-enhancing effects of estrogen, might also influence inflammation and depression outcomes [25]. In our study, we found that there was an increase in serum IL-1β levels in depressed patients; a similar observation was made in the previous study done by Yoshimura et al. [26]. Our findings corroborate these results; serum IL-1β levels were significantly higher in depressed patients (26.20±8.75 pg/ml) compared to the control group (17.71±7.15 pg/ml, p< 0.01). However, some studies report conflicting findings regarding the association between IL-1β and MDD [11,12]. This inconsistency could be due to varying psycho-neuro-inflammatory mechanisms across different populations. In the previous study done by Turnbull et al. [27] suggested that IL-1β activates the HPA axis, leading to the release of corticotropin-releasing hormone and adrenocorticotropic hormone, which indirectly contribute to depressive symptoms. Moreover, Goshen et al. [28] observed that IL-1β elevated the release of these hormones, supporting its role in depression. In the previous study done by Iwata et al. [29], further confirmed that elevated cytokine levels are major contributors to the development of depression, establishing a clear link between psychological stress and MDD.

From the present study, it was evident that there was a statistically significant (p<0.001) negative correlation between BDNF levels and HDRS scores with a Pearson coefficient (r) value of (-0.562) and a statistically significant (p<0.001) positive correlation between IL-1 β levels and HDRS scores with a Pearson coefficient (r) value of (+0.743). In some previous studies, it was found that there is a positive correlation between IL-1β and the severity of depression, which is in accordance with the present study [30]. Elevated cytokine levels, particularly IL-1β, are implicated in both the initiation and progression of depressive episodes. It was found in our study that serum BDNF levels in depressed patients with HDRS scores ≥ 15 were 3.42±1.26 ng/ml, which showed a statistically significant decrease (p<0.05) when compared to those with HDRS scores ≤ 15 of 4.64±1.48 ng/ml. Our study's strength lies in the inclusion of a comparison group and the estimation of serum BDNF and IL-1β levels in conjunction with HDRS scores, which provide a more robust assessment of depression severity. However, the study's limitations include its case-control design, small sample size, and the inability to assess the phenomenological aspects of depression and their relationship with inflammatory markers. Additionally, minor infections in some participants could have influenced the inflammatory markers, although we made efforts to exclude those with overt illnesses. Based on their medical history with overt illness, six patients were excluded to avoid bias in the study. Patients were screened with Mini-Cog, a mini-cognitive screening test, and patients with cognitive impairment were excluded. History of stressful events encountered by the case is ruled out by using HDRS rating scales accordingly, which may interfere with the biomarkers.

Future research should involve larger sample sizes, longitudinal studies, and the inclusion of patients with chronic medical conditions to allow for more comprehensive comparisons. Furthermore, the categorization of stressful life events in this study was somewhat arbitrary, and replication of these findings in different settings would help confirm the results.

## Conclusions

In this study, we explored the relationship between HDRS scores, serum BDNF, and IL-1β levels in individuals with depression from the southern part of India. Our findings suggest that elevated serum IL-1β levels and reduced BDNF levels are associated with an increased likelihood of developing depression. These results highlight the potential of using IL-1β and BDNF as biomarkers for assessing depression risk. However, to gain a deeper understanding of the biological mechanisms underlying the observed associations, further research involving larger, more homogeneous populations is essential.

## References

[1] Emon MP, Das R, Nishuty NL, Shalahuddin Qusar MM, Bhuiyan MA, Islam MR (2020). Reduced serum BDNF levels are associated with the increased risk for developing MDD: a case-control study with or without antidepressant therapy. BMC Res Notes.

[2] Mondal AC, Fatima M (2019). Direct and indirect evidences of BDNF and NGF as key modulators in depression: role of antidepressants treatment. Int J Neurosci.

[3] Kiecolt-Glaser JK, Derry HM, Fagundes CP (2015). Inflammation: depression fans the flames and feasts on the heat. Am J Psychiatry.

[4] Hannestad J, DellaGioia N, Bloch M (2011). The effect of antidepressant medication treatment on serum levels of inflammatory cytokines: a meta-analysis. Neuropsychopharmacology.

[5] Tong L, Prieto GA, Kramár EA, Smith ED, Cribbs DH, Lynch G, Cotman CW (2012). Brain-derived neurotrophic factor-dependent synaptic plasticity is suppressed by interleukin-1β via p38 mitogen-activated protein kinase. J Neurosci.

[6] Tolentino JC, Schmidt SL (2018). DSM-5 criteria and depression severity: implications for clinical practice. Front Psychiatry.

[7] Jeenger J, Singroha V, Sharma M, Mathur DM (2018). C-reactive protein, brain-derived neurotrophic factor, interleukin-2, and stressful life events in drug-naive first-episode and recurrent depression: a cross-sectional study. Indian J Psychiatry.

[8] Kudyar P, Gupta BM, Khajuria V, Banal R (2018). Comparison of efficacy and safety of escitalopram and vilazodone in major depressive disorder. Natl J Physiol Pharm Pharmacol.

[9] Arvind BA, Gururaj G, Loganathan S (2019). Prevalence and socioeconomic impact of depressive disorders in India: multisite population-based cross-sectional study. BMJ Open.

[10] Manidevi Karampudi, Shivprasad S, Sagar H, Bagewadi HG, Devendrappa BG, Shivalee A, Kallur R (2024). To evaluate the serum levels of suPAR and interlukin-6 and their associations to severity in chronic obstructive pulmonary disease patients: a case control study. Int J Pharm Clin Res.

[11] Charan J, Biswas T (2013). How to calculate sample size for different study designs in medical research?. Indian J Psychol Med.

[12] Zimmerman M, Martinez JH, Young D, Chelminski I, Dalrymple K (2013). Severity classification on the Hamilton Depression Rating Scale. J Affect Disord.

[13] Aggarwal K, Singh NR, Bala N, Singh M (2021). Major depressive disorder: association with vitamin C levels and role of vitamin C supplementation in pharmacotherapy. Int J Basic Clin Pharmacol.

[14] (2023). Windows | Epi InfoTM | Centers for Disease Control and Prevention (CDC). https://www.cdc.gov/epiinfo/pc.html.

[15] Farid Hosseini R, Jabbari Azad F, Talaee A (2007). Assessment of the immune system activity in Iranian patients with major depression disorder (MDD). Iran J Immunol.

[16] Yang K, Xie G, Zhang Z, Wang C, Li W, Zhou W, Tang Y (2007). Levels of serum interleukin (IL)-6, IL-1beta, tumour necrosis factor-alpha and leptin and their correlation in depression. Aust N Z J Psychiatry.

[17] Matthews KA, Schott LL, Bromberger JT, Cyranowski JM, Everson-Rose SA, Sowers M (2010). Are there bi-directional associations between depressive symptoms and C-reactive protein in mid-life women?. Brain Behav Immun.

[18] Molendijk ML, Spinhoven P, Polak M, Bus BA, Penninx BW, Elzinga BM (2014). Serum BDNF concentrations as peripheral manifestations of depression: evidence from a systematic review and meta-analyses on 179 associations (N=9484). Mol Psychiatry.

[19] Karege F, Perret G, Bondolfi G, Schwald M, Bertschy G, Aubry JM (2002). Decreased serum brain-derived neurotrophic factor levels in major depressed patients. Psychiatry Res.

[20] Lee BH, Kim H, Park SH (2007). Decreased plasma BDNF level in depressive patients. J Affect Disord.

[21] Madsen CA, Navarro ML, Elfving B, Kessing LV, Castrén E, Mikkelsen JD, Knudsen GM (2024). The effect of antidepressant treatment on blood BDNF levels in depressed patients: a review and methodological recommendations for assessment of BDNF in blood. Eur Neuropsychopharmacol.

[22] Maes M, Meltzer HY, Buckley P, Bosmans E (1995). Plasma-soluble interleukin-2 and transferrin receptor in schizophrenia and major depression. Eur Arch Psychiatry Clin Neurosci.

[23] Brietzke E, Stertz L, Fernandes BS (2009). Comparison of cytokine levels in depressed, manic and euthymic patients with bipolar disorder. J Affect Disord.

[24] Tubbs JD, Ding J, Baum L, Sham PC (2020). Immune dysregulation in depression: evidence from genome-wide association. Brain Behav Immun Health.

[25] Roved J, Westerdahl H, Hasselquist D (2017). Sex differences in immune responses: hormonal effects, antagonistic selection, and evolutionary consequences. Horm Behav.

[26] Yoshimura R, Katsuki A, Atake K, Hori H, Igata R, Konishi Y (2017). Influence of fluvoxamine on plasma interleukin-6 or clinical improvement in patients with major depressive disorder. Neuropsychiatr Dis Treat.

[27] Turnbull AV, Rivier CL (1999). Regulation of the hypothalamic-pituitary-adrenal axis by cytokines: actions and mechanisms of action. Physiol Rev.

[28] Goshen I, Yirmiya R (2009). Interleukin-1 (IL-1): a central regulator of stress responses. Front Neuroendocrinol.

[29] Iwata M, Ota KT, Duman RS (2013). The inflammasome: pathways linking psychological stress, depression, and systemic illnesses. Brain Behav Immun.

[30] Zou W, Feng R, Yang Y (2018). Changes in the serum levels of inflammatory cytokines in antidepressant drug-naïve patients with major depression. PLoS One.

